# Synergistic activity of Pitstop-2 and 1,6-hexanediol in aggressive human lung cancer cells

**DOI:** 10.1186/s11671-025-04184-z

**Published:** 2025-01-21

**Authors:** Sílvio Terra Stefanello, Caren Rigon Mizdal, Aline Franzen da Silva, Luca Matteo Todesca, Félix Alexandre Antunes Soares, Victor Shahin

**Affiliations:** 1 Institute of Physiology II, University of Münster, Robert-Koch-Str. 27b, 48149 Münster, Germany; 2 Department of Biochemistry and Molecular Biology, Federal University of Santa Maria, Av. Roraima 1000, Santa Maria, RS 97105-900 Brazil

**Keywords:** 1,6-hexanediol, *Caenorhabditis elegans*, Erlotinib resistance, NSCLC, Pitstop-2

## Abstract

Metastatic cancer cells undergo metabolic reprogramming, which involves changes in the metabolic fluxes, including endocytosis, nucleocytoplasmic transport, and mitochondrial metabolism, to satisfy their massive demands for energy, cell division, and proliferation compared to normal cells. We have previously demonstrated the ability of two different types of compounds to interfere with linchpins of metabolic reprogramming, Pitstop-2 and 1,6-hexanediol (1,6-HD). 1,6-HD disrupts glycolysis enzymes and mitochondrial function, enhancing reactive oxygen species production and reducing cellular ATP levels, while Pitstop-2 impedes clathrin-mediated endocytosis and small GTPases activity. Besides, both compounds interfere with the integrity of nuclear pore complexes, the gatekeepers for all nucleocytoplasmic transport. Herein, we investigate the possible synergistic effects of both compounds on lowly, highly metastatic, and erlotinib-resistant non-small cell lung cancer. We observe a synergistic cytotoxic effect on erlotinib-resistant cells. Moreover, motility assays show that the compounds combination significantly impedes the motility of all cells. Drug safety and tolerability assessments were validated using the in vivo model organism *Caenorhabditis elegans*, where fairly high doses showed negligible impact on survival, development, or behavioral parameters. Our findings propose that the 1,6-HD and Pitstop-2 combination may usher in the design of potent strategies for treating advanced lung cancer.

**Supplementary Information:**

The online version contains supplementary material available at 10.1186/s11671-025-04184-z.

## Introduction

Cancer remains one of the most formidable health challenges of our time, defined by its vast complexity and diversity. Fundamental to the nature of cancer are the ‘hallmarks of cancer’, first coined by Hanahan and Weinberg, which include sustaining proliferative signaling, evading growth suppressors, resisting cell death, enabling replicative immortality, inducing angiogenesis, activating invasion and metastasis, reprogramming energy metabolism, and evading immune destruction [1–4]. Metabolic reprogramming alters the cellular bioenergetic landscape necessary for supporting the unusual growth and survival needs of cancer cells [5]. Hence, glycolysis enzymes and mitochondria are among the key players in malignantly transformed cancer cells, and present potent targets for cancer treatment strategies [6–8]. Another key player in malignant cancer cells are nuclear pore complexes (NPCs), which mediate all nucleocytoplasmic transport. Aggressive cancer cells manipulate NPCs to substantially elevate the nucleocytoplasmic transport rates, and targeting NPC transport has consequentially become a highly desired pharmacological approach for novel anti-cancer strategies [9, 10]. By targeting the metabolic flexibility that cancer cells exhibit, it may be possible to disrupt the homeostatic mechanisms they rely on, leading to a reduction in their proliferation and survival [11]. This approach is particularly attractive because it may be less prone to resistance mechanisms that cancer cells develop against conventional cytotoxic therapies.

In our recent study, 1,6-hexanediol (1,6-HD) has emerged as a compound of interest due to its inhibitory activity on enzymes crucial for glycolysis, such as lactate dehydrogenase A (LDHA) [12], the major player for ATP production in the Warburg effect of cancer cells [2, 13]. Moreover, we observed that 1,6-HD disrupts NPCs and mitochondrial integrity, eventually affecting the viability and preventing the migratory activity of highly metastatic non-small cell lung cancer (NSCLC) cells (A549_3R) [12, 14, 15]. On the other hand, we observed that Pitstop-2, a potent inhibitor of clathrin-mediated endocytosis (CME), reduces cellular motility [16], a critical factor for the invasion and metastasis of cancer cells. The CME-independent activities of Pitstop-2 rely strongly on its disruptive effects on NPCs and the small GTPase Ran (essential for nucleocytoplasmic transport) [17], and Rac1 (essential for overall motility) [18, 19].

In the present study, we have combined 1,6-HD and Pitstop-2 to assess the synergistic effects on NSCLC cells at varying stages of malignancy, encompassing low metastatic potential (A549_0R), high metastatic rate (A549_3R), and resistance to the targeted therapy erlotinib (A549_3Rres). Additionally, we evaluated the drug safety and potential cytotoxicity of this combination of compounds in the model organism *Caenorhabditis elegans* (*C. elegans*) to ascertain the safety profile of the therapy.

## Materials and methods

### Cell culture

A549 lung adenocarcinoma were cultured were cultured at 37 °C, 5% CO_2_ in Dulbecco’s modified Eagle’s medium (Invitrogen Corp., Karlsruhe, Germany) supplemented with 10% fetal calf serum (FCS, PAA Clone, Coelbe, Germany). A549 cell line was generously provided by the Institute of Physiology II (Münster, Germany). The generation of the highly aggressive A549 cell line (A549_3R), was described previously [20]. A549_3R resistant against erlotinib were obtained as described previously [21]. Pitstop-2, 1,6-HD, erlotinib and EGF were purchase from Sigma-Aldrich (St. Louis, MO, USA).

### Cytotoxicity measurement

A549_0R and A549_3R were grown at 5 × 10^3^ cells/well in 96-well culture plates and treated with different concentrations of 1,6-HD and Pitstop-2 in combination. CCK8 reagent was added to each well, the plate was incubated for 1 h and absorbance values were determined at 450 nm.

Effects of the combination of 1,6-HD and Pitstop-2 were assessed by the fractional inhibitory concentration index (FICI) and made isobolograms (Figure S1). FICI was calculated using the following formula:

$${\text{FIC}}_{{\text{A}}} = \, \left( {{\text{IC}}_{{{5}0}} {\text{of Drug A in combination}}/{\text{IC}}_{{{5}0}} {\text{of Drug A alone}}} \right)$$

$${\text{FIC}}_{{\text{B}}} = \, \left( {{\text{IC}}_{{{5}0}} {\text{of Drug B in combination}}/{\text{IC}}_{{{5}0}} {\text{of Drug B alone}}} \right)$$

$${\text{FICI }} = {\text{ FIC}}_{{\text{A}}} + {\text{ FIC}}_{{\text{B}}}$$

FICI < 0.5, 0.5 ≤ FICI ≥ 1, 1 < FICI ≤ 4, and FICI > 4 were defined as synergy, additive effect, indifference, and antagonism, respectively [22].

### Cell motility analysis

The assay was prepared according to our previous publication [14]. Prior to the start of image recording with time-lapse video microscopy, A549_3R were exposed to the combination of 1% 1,6-HD and 30 µM of Pitstop-2 for 30 min. After washout, the flasks were transferred to heating chambers (37 °C) on the microscopes (Zeiss Axiovert 40C). The cells were imaged in 600 s intervals for 10 h controlled by HiPic 32 or WASABI software (Hamamatsu).

### *C. elegans* strain, maintenance, and treatment

The *C. elegans* strain used in this study was Bristol N2 (wild-type) obtained from the Caenorhabditis Genetics Center (CGC, University of Minnesota, Minneapolis, MN, USA). For the assay, gravid hermaphrodite were synchronized through a bleaching protocol using bleaching solution (1% NaOCl, 0.25 M NaOH) that consists of breaking the animal’s cuticle, releasing the eggs in the middle by isolating embryos from gravid hermaphrodites. Eggs were left in M9 buffer for overnight to allow all viable eggs to hatch and reach the first larval stage L1.

### Survival rate

L1 worms was transferred to M9 buffer (42 mM Na_2_HPO_4_, 22 mM KH_2_PO_4_, 8.5 mM NaCl and 1 mM MgSO_4_) in presence of the 1,6-HD and Pitstop-2 in combination for one hour. After incubation, the survival rate was evaluated in approximately 100 worms per condition and the remaining worms were seeded with *Escherichia coli* OP50 as a food source, at 20 °C until the worms achieve the adult stage at a temperature of 20 °C [23]. Behavioral analyzes were carried out with individuals in adulthood stage.

### Pharyngeal pumping

Using a microscope, the number of pharyngeal contractions of each worm on the maintenance plate was counted for 10 s; the tests were run in triplicate. Subsequently, the values for each worm were averaged and expressed as the pharyngeal pumping/minute [24]. Three assays were carried out at different times, and five worms were analyzed in each experiment.

### Head thrashes

Previously treated worms in the young adult stage were chosen at random and placed for 1 min in a drop of M9 buffer. Then, the number of head movements of each worm was counted for 20 s. Three independent experiments were carried out, with a total of 10 worms per group per experiment [25].

### Touch response

To analyze the touch response, we used N2 strain. The behavior was assessed by gently touching the head region of the nematode with a bristle brush and counting how many times worms responded to ten touches with a 10-s rest period between trials [26]. Three assays were carried out at different times, and five worms were analyzed in each experiment.

### Statistical analysis

Each experimental condition was repeated at least three times. Data are presented as mean values ± standard error of the mean (S.E.M). Results are considered as statistically significant at the probability level *P* < 0.05, using GraphPad Prism 9.0.0 (GraphPad Software, Inc). The details regarding the number of experiments and analyzed cells, applied statistical tests and *P* values are specified in the corresponding parts.

## Results and discussion

### Combination of 1,6-HD and Pitstop-2 severely harms NSCLC

Remarkable increase of the rate of endocytosis and nucleocytoplasmic transport, and substantial changes in metabolic activities are associated with advanced stages of malignancy [3, 27].

In the present report we reasoned that a combination of 1,6-HD and Pitstop-2 spectrum of activities may severely harm cancer cells owing to their strong dependence on the pharmacological targets of the two compounds. We performed acute exposure experiments of lowly metastatic (A549_0R), highly metastatic (A549_3R), and erlotinib-resistant (A549_3Rres) NSCLC cells to different concentrations of the combination of 1,6-HD and Pitstop-2.

**Fig. 1:**
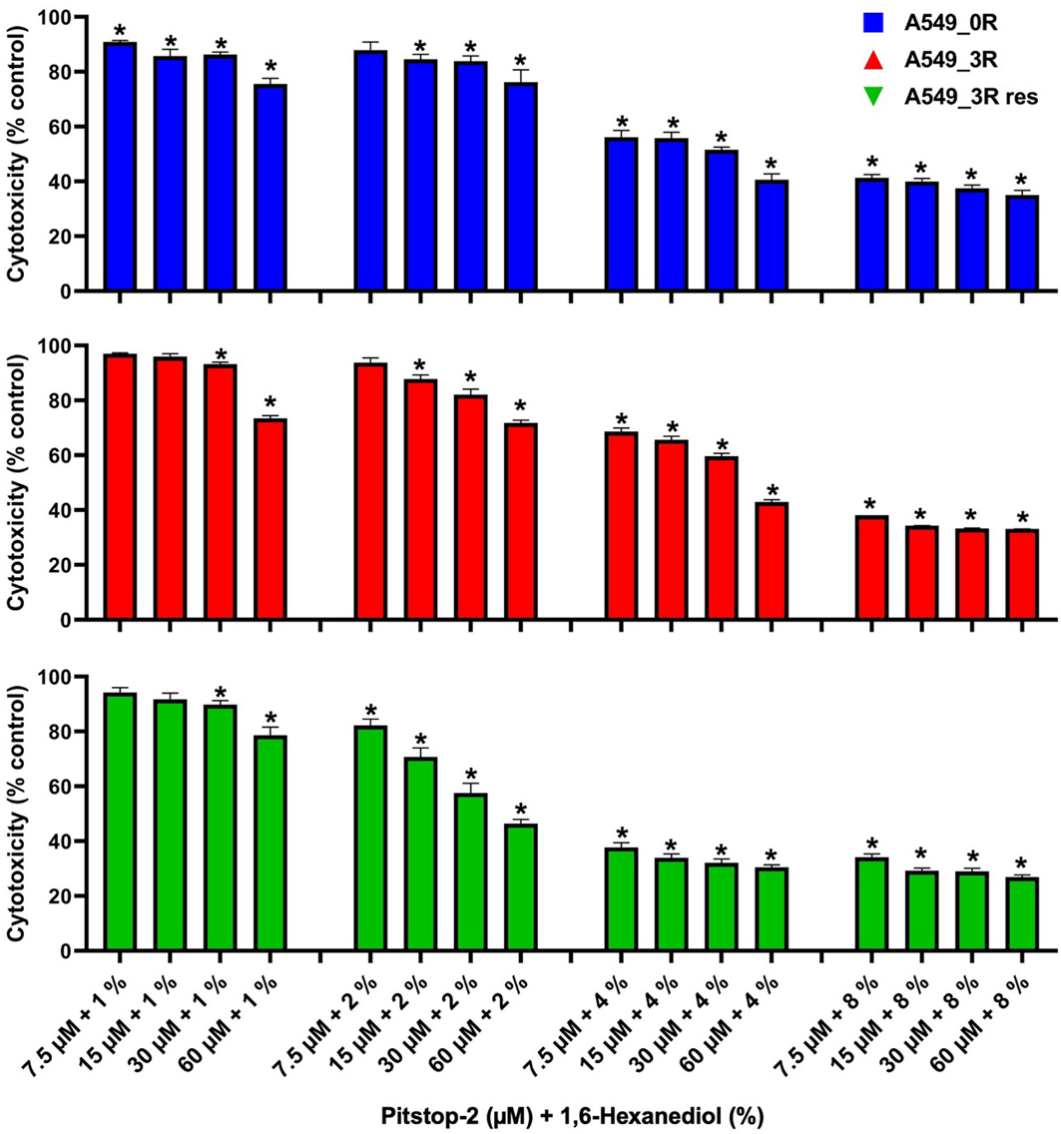
Cytotoxicity testing of the combination of 1,6-HD and Pitstop-2 in lowly, highly metastatic, and erlotinib-resistant NSCLC (A549_0R, A549_3R and A549_3Rres, respectively). The CCK-8 assay was used, which measures cell viability after one hour of exposure to a range of concentration of Pitstop-2 and 1,6-HD in combination by correlating the production of colored formazan dye to the amount of living cells in culture. N = 3. Asterisks indicate a statistically significant difference, *P* < 0.05, performed by One-way ANOVA (Bonferroni multiple comparison test)

We tested several combinations involving concentrations of (1%, 2%, 4%, and 8%) for 1,6-HD and (7.5 µM, 15 µM, 30 µM, and 60 µM) for Pitstop-2. All NSCLC tested showed a reduction of approximately 20% in viability when exposed to the combination that contained the lowest concentration of 1,6-HD used (1%) with the highest of Pitstop-2 (60 µM) (Fig. 1). Hence, there is a strong synergistic effect of 1,6-HD and Pitstop-2 on NSCLC. Interestingly, A549_3Rres cells showed greater vulnerability to a combination of 1,6-HD and Pitstop-2. Furthermore, the synergistic combination of 2% of 1,6-HD and 15 µM of Pitstop-2 proved to be the cytotoxic effect selective for A549_3Rres, based on our assessment of the fractional inhibitory concentration index (FICI), which is used in pharmacological research to determine the interaction of drugs in combination (Table 1) [22].

**Table 1:** Assessment of the fractional inhibitory concentration index (FICI) of the combination of 1,6-hexanediol (1,6-HD) and Pitstop-2 (P2) in NSCLC

	P2 (µM)	1,6-HD (%)	FIC P2	FIC 1,6-HD	FICI	Effect
A549_3Rres	15	2	0.125	0.25	0.375	Synergy
A549_3R	60	4	0.5	0.5	1	Additive
A549_0R	60	2	0.5	0.25	0.75	Additive

This is a promising observation as developing resistance to erlotinib in the treatment of NSCLC is a well-documented and prevalent problem [28, 29]. Therefore, our results propose that a combination of 1,6-HD and Pitstop-2 with erlotinib may prove to be a potent anti-cancer strategy minimizing the development of drug resistance and improving the prognostic outcomes in lung cancer therapy.

### Combination of 1,6-HD and Pitstop-2 in NSCLC motility

**Fig. 2:**
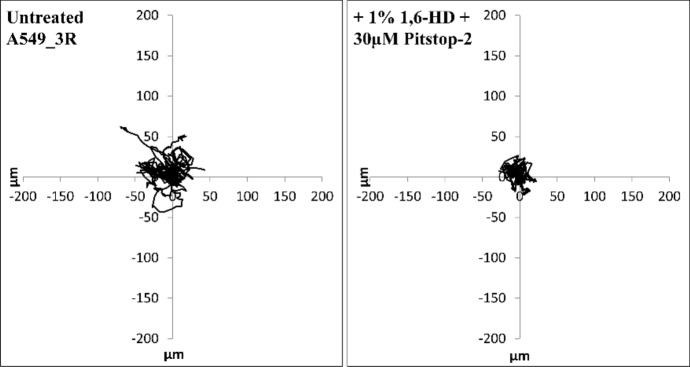
Spider plots for random migration tracks and cell motility analysis of highly metastatic lung cancer cells (A549_3R) after exposure to the combination of 1% 1,6-HD and 30 µM of Pitstop-2. N = 3, 20 cells each condition

Cell motility is pivotal for cancer cells metastasis. To validate whether the 1,6-HD and Pitstop-2 affect cancer cells motility, we exposed A549_3R cells for 30 min to concentrations of 1% 1,6-HD and 30 µM Pitstop-2 in combination and separately. After removal of the compounds, we used time-lapse video microscopy to observe the cells over a 10 h interval (Fig. 2 and Videos S1-S4), and next analyzed the velocity, travelled distance and structural index of cells (Table 2). Cells treated with either 1% 1,6-HD (Video S2) or 30 µM Pitstop-2 (Video S3) showed some impairment in motility over 10 h compared to untreated control cells, which was most pronounced at the beginning of the video-microscopy recording, but they eventually recovered towards the end of the recording time (Video S1). This observation suggests acute on-time exposure of A549_3R cells to either 30 µM Pitstop-2 or 1% 1,6-HD reversibly hinders their ability to migrate, indicating that the drugs’ effects on motility may require either a higher concentration, prolonged exposure time or frequency to manifest itself. Cells exposed to 1% 1,6-HD exhibited a morphological transformation to a rounded shape yet maintained their capacity for movement albeit less effectively compared to untreated cells (Video S2). This suggests that while 1,6-HD impacts the structural integrity of cells, it does not entirely abolish cellular motility. The structural changes of cancer cells caused by 1,6-HD may be caused by its disruption of the bonds between NPCs and cytoskeletal elements as discussed in our recent work [14]. The combined treatment using 1% 1,6-HD and 30 µM of Pitstop-2 resulted in altered cell shape and reduced motility (Video S4). This synergistic effect implies that while each compound alone may only partially affect the cells, their combination potentiates the disruption of cell motility mechanisms. We assume that the observed morphological changes and reduced motility result from the collective actions of 1,6-HD and Pitstop-2 in combination on the NPCs, CME, mitochondrial integrity, LDH-A and the activity of the small GTPases Ran and Rac1. The targets of the two compounds are pivotal in all types of cancer, which proposes the combination of the two compounds as a possible anti-cancer strategy for intra-tumoral applications to limit the side effects.

**Table 2:** Analysis of A549_3R cells migratory behavior and morphology 10 h after exposure to the combination of 1% 1,6-HD and 30 µM Pitstop-2. Data are shown as the mean±S.E.M. Asterisks indicate a statistically significant difference, P<0.05, performed by t-test

	Untreated A549_3R	+ 1% 1,6-HD + 30 µM Pitstop-2
Structural index at 10 h	0.67 ± 0.04	0.38 ± 0.03*
Travelled distance (µm)	35.56 ± 6.35	16.42 ± 2.32*
Velocity (µm/min)	0.32 ± 0.04	0.18 ± 0.02*

### Combination of 1,6-HD and Pitstop-2 activity in *C. elegans* survival and behavior assays

*C. elegans* (N2 Bristol, wild type) is widely used as an animal model for diverse diseases including cancer [30], and model organism for initial testing of drug efficacy, safety and toxicity [31, 32]. The extensive utility of this model in toxicology and pharmacology arises from its remarkable capacity for screening and evaluating the toxicological profiles of novel compounds [33–35].

**Fig. 3:**
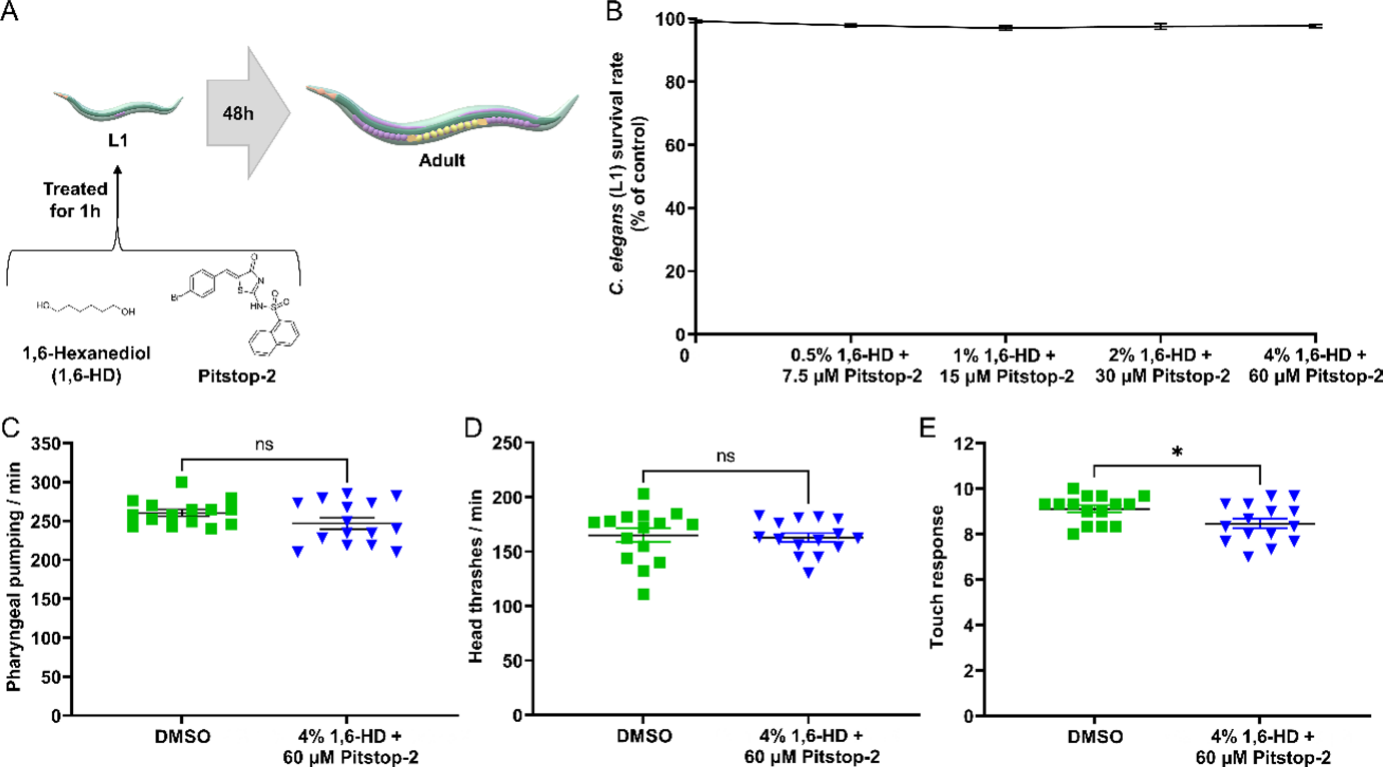
Assessment of the drug safety of 1,6-HD and Pitstop-2 in the model organism *C. elegans.* **A** Schematic illustration of the treatment of *C. elegans* with 1,6-HD and Pitstop-2. **B** Survival rate of *C. elegans* larvae (stage L1) after 1 h exposure to different concentrations of the combination of 1,6-HD and Pitstop-2. **C** Pharyngeal pumping rate of adult *C. elegans* per minute. **D** Number of times adult *C. elegans* moves its head per minute. **E** The touch response test of adult *C. elegans* touch response. N = 3, and 15 worms each. Asterisks indicate a statistically significant difference, *P* < 0.05, performed by *t*-test

Therefore, we applied a protocol for exposing animals in the larval stage (L1), the most vulnerable, to different concentrations of the combination of 1,6-HD and Pitstop-2 (0.5% + 7.5 µM, 1% + 15 µM, 2% + 30 µM and 4% + 60 µM) (Fig. 3A). As shown in Fig. 3, we observed that exposing the animals for one hour did not cause any decrease in the survival rate (Fig. 3B), even at concentrations well above those that disrupt the nuclear pore complex and inhibit the activity of small GTPases, 1,6-HD, and Pitstop-2, respectively. To assess whether exposure to this combination did not promote any changes in development or compromise any behavioral parameters of the living organism, they were allowed to develop until adulthood when the tests were carried out.

We demonstrated that the combination of 1% 1,6-HD and 30 µM Pitstop-2 promotes severe effects on A549_3R motility (Fig. 2). However, in *C. elegans*, even when tested at substantially higher concentrations (4% 1,6-HD and 60 µM Pitstop-2), the motility parameter, head thrashes (Fig. 3C), did not show any changes. We also evaluated a parameter that is usually impacted under stress conditions and can be influenced depending on the metabolic state of the worm, which is the pharyngeal pumping. Besides, the pharyngeal pumping assay is used as a readout to assess the effects of experimental conditions, such as exposure to toxins, on these worms’ overall health and behavior. Figure 3C demonstrates that the combination of 4% 1,6-HD and 60 µM Pitstop-2 did not promote any change in the action of the pharynx and that acute exposure to the compound’s combination could not cause any long-term damage.

Interestingly, as shown in Fig. 3E, we observed a slight but significant decrease in the touch response, demonstrating that the combination of 4% 1.6-HD and 60 µM Pitstop-2 interfered with the mechanosensory mechanism of *C. elegans*. As the mechanosensory mechanism of *C. elegans* is regulated by small GTPases, particularly RHO-1, the effect only corroborates the previously mentioned effects resulting from the action that Pitstop-2 exerts on these proteins.

Therefore, our data obtained in the *C. elegans* model cautiously propose that the combination of 1,6-HD and Pitstop-2 may be safe to use in future.

## Conclusion

The current study presents compelling evidence that the combination of 1,6-hexanediol (1,6-HD) and Pitstop-2 exhibits a potent synergistic effect on non-small cell lung cancer (NSCLC) cells, particularly enhancing the cytotoxicity against erlotinib-resistant variants. The findings indicate that this combination reduces cell viability and impairs cellular motility, a critical factor in cancer metastasis. Notably, the combination therapy appears to selectively target cancer cells while sparing normal cells, as demonstrated by the lack of adverse effects in C. elegans, suggesting a favorable therapeutic index. These results advocate for the potential of 1,6-HD and Pitstop-2 as promising anti-cancer strategies. Furthermore, future investigations will focus on developing nanoparticles combining 1,6-HD and Pitstop-2 to improve the specificity of drug delivery to cancer cells and reduce potential effects on normal cells.

## Supplementary Information

Additional file1

Additional file2

Additional file3

Additional file4

Additional file5

## Data Availability

All data generated or analyzed during this study are included in this published article and its supplementary information files. Our study utilized the model organism Caenorhabditis elegans, a nematode, which is not classified as a higher invertebrate. Therefore, no approval or consent was required for the experiments conducted.
