# Supplementary material for: Synergistic activity of Pitstop-2 and 1,6-hexanediol in aggressive human lung cancer cells
Source: Discov Nano. 2025 Jan 21;20(1):12. doi: 10.1186/s11671-025-04184-z (PMC11751257; doi:10.1186/s11671-025-04184-z)
Supplement: Supplementary file 1 — Additional file1 [file 11671_2025_4184_MOESM1_ESM.docx]

# Supporting Information

Synergistic anti-cancer activity of Pitstop-2 and 1,6-Hexanediol in aggressive human lung cancer cells

Sílvio Terra Stefanello^1^, Caren Rigon Mizdal^1^, Aline Franzen da Silva^2^, Luca Matteo Todesca^1^, Félix Alexandre Antunes Soares^2^, Victor Shahin^1^*

^1^ Dr. S. T. Stefanello, Dr. C. R. Mizdal, Dr. L. M. Todesca, and Prof. Dr. V. Shahin

Institute of Physiology II

University of Münster

Robert-Koch-Str. 27b, 48149 Münster, Germany.

^2^ A. F. da Silva and Prof. Dr. F. A. A. Soares

Department of Biochemistry and Molecular Biology

Federal University of Santa Maria

Av. Roraima 1000, 97105-900 Santa Maria, RS, Brazil

*** Corresponding Authors:**

Prof. Dr. Victor Shahin

E-mail: [shahin@uni-muenster.de](mailto:shahin@uni-muenster.de)


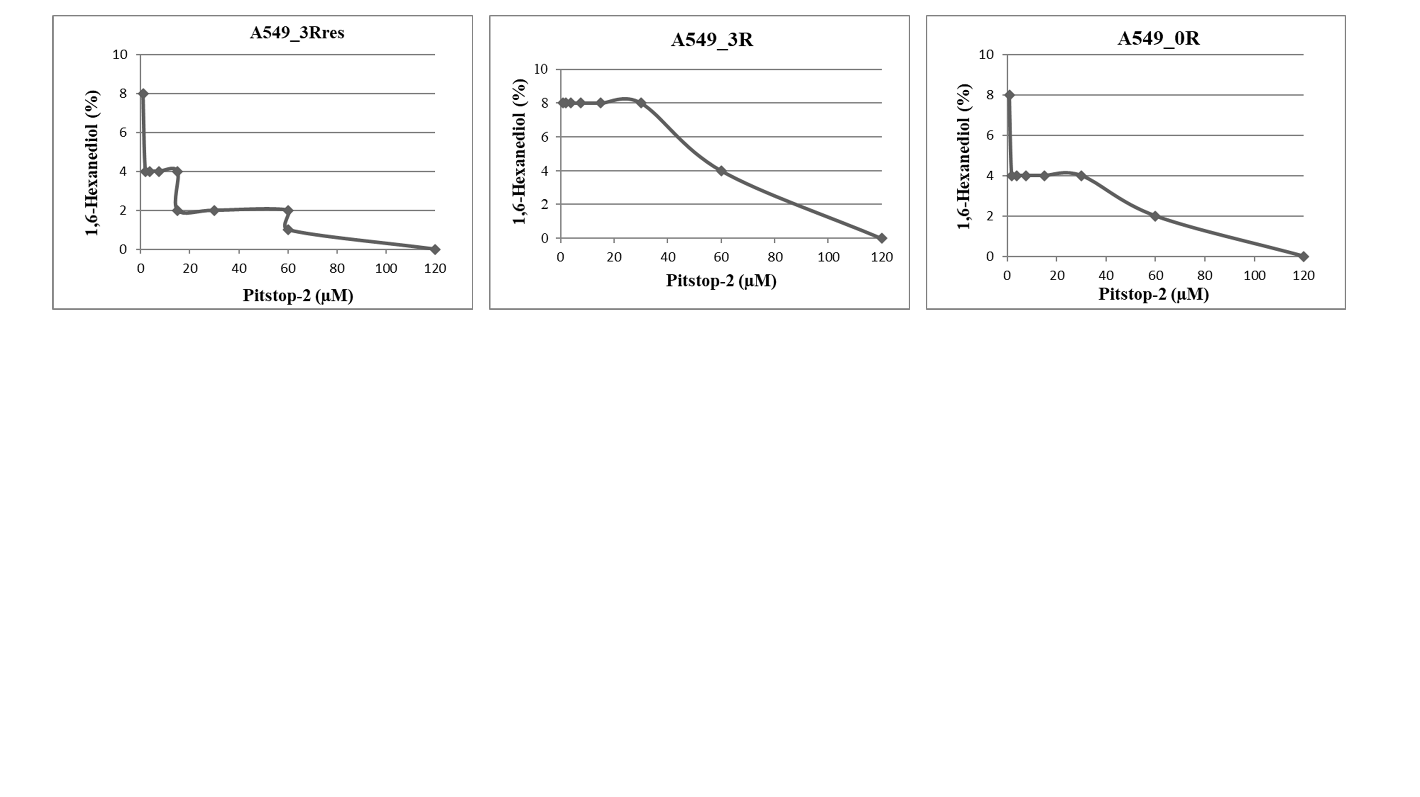


**Fig. S1** Isobolograms of 1,6-HD and Pitstop-2 in combination activity against lowly metastatic (A549_0R), highly metastatic (A549_3R), and erlotinib-resistant (A549_3Rres) NSCLC cells.

# Supporting movie captions:

## Video S1 Time-lapse video microscopy of the motility of highly metastatic human lung cancer cells (A549_3R), observed for 10h on 2D collagen matrix.

## Video S2 Time-lapse video microscopy of the motility of highly metastatic human lung cancer cells (A549_3R), observed for 10h on 2D collagen matrix, after 30 minutes exposure time to, followed by removal of 1% 1,6-HD.

## Video S3 Time-lapse video microscopy of the motility of highly metastatic human lung cancer cells (A549_3R), observed for 10h on 2D collagen matrix, after 30 minutes exposure time to, followed by removal of 30µM of Pitstop-2.

## Video S4 Time-lapse video microscopy of the motility of highly metastatic human lung cancer cells (A549_3R), observed for 10h on 2D collagen matrix, after 30 minutes exposure time to, followed by removal of a combination of 1% 1,6-HD and 30µM of Pitstop-2.
